# Comparison over Time of Adverse Drug Reactions in Diabetes Patients Treated with Sodium-Glucose Cotransporter 2 Inhibitors

**DOI:** 10.24546/0100490464

**Published:** 2024-07-25

**Authors:** MAI UEDA, MASAKO ZENIBAYASHI, TOMOKO YAMADA, SHUN-ICHIRO ASAHARA, WATARU OGAWA

**Affiliations:** 1 Department of Diabetes and Endocrinology, Yodogawa Christian Hospital, Osaka, Japan; 2 Division of Diabetes and Endocrinology, Department of Internal Medicine, Kobe University Graduate School of Medicine, Kobe, Japan

**Keywords:** Diabetes mellitus, Sodium-glucose cotransporter 2 inhibitor, Adverse drug reaction

## Abstract

**BACKGROUNDS:**

The prescription of sodium-glucose cotransporter-2 (SGLT2) inhibitors have been increasing due to their additional benefits, including weight loss, cardioprotection and renoprotection. Accordingly, there are concerns about the potential rise in severe adverse drug reactions (ADRs), such as urinary tract infections, diabetic ketoacidosis, volume depletion, and hypoglycemia. The Society has announced recommendations on the proper use of SGLT2 inhibitors. We aimed to elucidate the recent occurrence of severe ADRs which need discontinuation of SGLT2 inhibitors or hospitalization.

**METHODS:**

In this retrospective cohort study, we identified 391 diabetic patients who were prescribed SGLT2 inhibitors upon admission to our hospital between April 2017 and March 2023. Of these, 68 patients who discontinued SGLT2 inhibitors for reasons other than ADRs were excluded. Patients were classified into the 2017 group and the 2020 group based on the treatment period of SGLT2 inhibitors, and the occurrence of ADRs and patient backgrounds were compared between the two groups.

**RESULTS:**

A total of 323 eligible patients were identified. Discontinuations of SGLT2 inhibitors decreased in the 2020 group (*p* < 0.05). However, discontinuations due to frailty increased (*p* < 0.05). Hospitalization due to ADRs, specifically those due to urinary tract infections, diabetic ketoacidosis, or volume depletion, did not specifically decrease (*p* = 0.273).

**CONCLUSIONS:**

This study indicated that there has been some improvement in the awareness of the proper use of SGLT2 inhibitors and there is still a need to continue enlightenment activities.

## INTRODUCTION

Sodium-glucose cotransporter-2 (SGLT2) inhibitors show multifaceted metabolic improvement effects such as weight loss, improvement of blood pressure, lipids, and fatty liver in addition to hypoglycemic effects. Furthermore, cardioprotective and renoprotective effects have been reported in a series of large-scale clinical trials in recent years (EMPA-REG OUTCOME, DECLARE-TIMI 58, CANVAS, DAPA-CKD, CREDENECE, EMPEROR-Reduced, EMPEROR-Preserved, DAPA-HF, DELIVER) (1–9), and the patients treated with SGLT2 inhibitors is rapidly increasing.

On the other hand, however, adverse drug reactions (ADRs) such as ketoacidosis, urinary tract infections, and dehydration had been reported (10–12). The first SGLT2 inhibitor was launched in 2014 and other several SGLT2 inhibitors have followed in the next few years. Whereas the Japan Diabetes Society and others have issued a warning against ADRs from 2014 (13), we have experienced a certain number of cases which were not complied with proper use of SGLT2 inhibitors in clinical practice. In this study we aimed to determine whether the occurrence and severity of ADRs improved over time.

Therefore, we retrospectively investigated the patients with a history of SGLT2 inhibitor prescriptions at our hospital and evaluated the case with discontinuation of SGLT2 inhibitors regarding with cause ADRs, severity, and the clinical backgrounds. We believe that validation in acute care hospitals will help us understand the current clinical practice in real-world about SGLT2 inhibitor ADRs.

## MATERIALS AND METHODS

### Participants and study design

**Figure 1:**
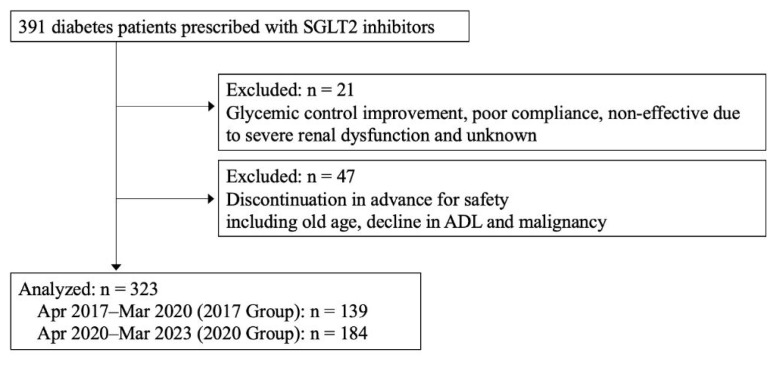
Flow chart of participant enrolment

We selected 391 diabetic patients with a history of SGLT2 inhibitor prescription between April 2017 and March 2023, who were treated in diabetes and endocrinology at the time of admission to our hospital. Of these, 21 patients discontinued SGLT2 inhibitors for reasons other than ADRs (improvement of blood glucose, poor compliance, ineffectiveness due to severe renal function decline, or reason for discontinuation unknown), 47 patients discontinued in advance due to the risk of developing ADRs based on patient backgrounds (elderly, decline in ADL, malignant tumor). Thus, a total of 68 patients were excluded, leaving 323 patients for analysis (Figure 1).

SGLT2 inhibitor have been granted approval for use in patients with heart failure or renal failure, leading to an increase in prescriptions of SGLT2 inhibitors since 2020 in Japan. We anticipated that the backgrounds of patients prescribed SGLT2 inhibitors would change after 2020. Therefore, we classified into two groups: one for the 3-year period from April 2017 to March 2020 (2017 group) and the other for the 3-year period from April 2020 to March 2023 (2020 group). A total of 139 and 184 patients were included in each group, respectively (Figure 1). We retrospectively explored the patients who discontinued SGLT2 inhibitors due to ADRs in each period, and then compared the causes of SGLT2 inhibitor discontinuation and clinical backgrounds.

### Data collection

The study retrospectively collected patient information on age at admission, duration of diabetes, height and weight, complications (diabetic nephropathy, diabetic retinopathy, coronary artery disease and cerebrovascular disease), history of heart failure and hypertension, medication history (hypoglycemic drugs, insulin injections, antihypertensive drugs and steroids), blood tests and urine analysis medical records. Information of drinking habits, activities of daily living (ADL) decline, cognitive decline, diaper wearing and urinary catheter placement were also collected, which are as risks for ADRs. Habitual drinkers were defined as having a drinking habit (occasional drinking was excluded). Patients who were certified for long-term care or had a level of care equivalent to that required were defined as having decreased ADLs. Cognitive decline was defined as having cognitive decline or dementia documented in the medical records. The causes of SGLT2 inhibitors discontinuation were collected from the medical history chart. ADRs for SGLT2 inhibitors included urinary tract/genital infections, polyuria, diabetic ketoacidosis, volume depletion, hypoglycemia, stroke, and frailty. Frailty was diagnosed according to the definition of Fried et al. proposed by the Japan Geriatrics Society (14).

### Ethics

This study was approved by the Ethics Committee of Yodogawa Christian Hospital (No. 2023-022) and was conducted in accordance with the Declaration of Helsinki.

### Statistical analysis

Data were presented as mean ± SD (%) for normally distributed data, the median (interquartile range) for nonnormally distributed data, or n values (%) for categorical data. Intergroup differences of normally or nonnormally distributed data were tested for significance with the unpaired Student’s t test or Mann–Whitney U test, respectively. For categorical data, Fisher’s exact test was applied. A P value of <0.05 was considered statistically significant. All statistical analysis were performed with EZR version 1.52 (Saitama Medical Center, Jichi Medical University, Saitama, Japan), which is a graphical user interface for R version 4.02 (The R Foundation for Statistical Computing, Vienna, Austria). More precisely, it is a modified version of R commander designed to add statistical functions frequently used in biostatistics (15).

## RESULTS

The clinical backgrounds of patients who had received SGLT2 inhibitors in our hospital between April 2017 and March 2023 on admission was as follows: mean age 62.6 years, median disease duration 8 years, mean body mass index 27.5 kg/m^2^, median HbA1c 8.8%, median eGFR 72.7 mL/min per 1.73m^2^ (Table I). Only 2 patients (0.6%) had type 1 diabetes, the remaining patients had type 2 diabetes. Comparing the 2017 group and the 2020 group, median age was older and median HbA1c is lower in the 2020 group. Complications and pre-existing conditions were not significantly different between the two groups.

The study of diabetes medications revealed that dipeptidy-l peptidase 4 inhibitors were the most prescribed drugs (214 patients, 66.3%), followed by biguanides (168 patients, 52.0%), and insulin (71 patients, 22.0%) (Table II). ACE-I/ARBs were the most common medications for hypertension and heart failure (143 patients, 44.3%), followed by Ca antagonists (131 patients, 40.6%). There were no significant differences in medication use between the 2017 group and the 2020 group.

**Figure 2:**
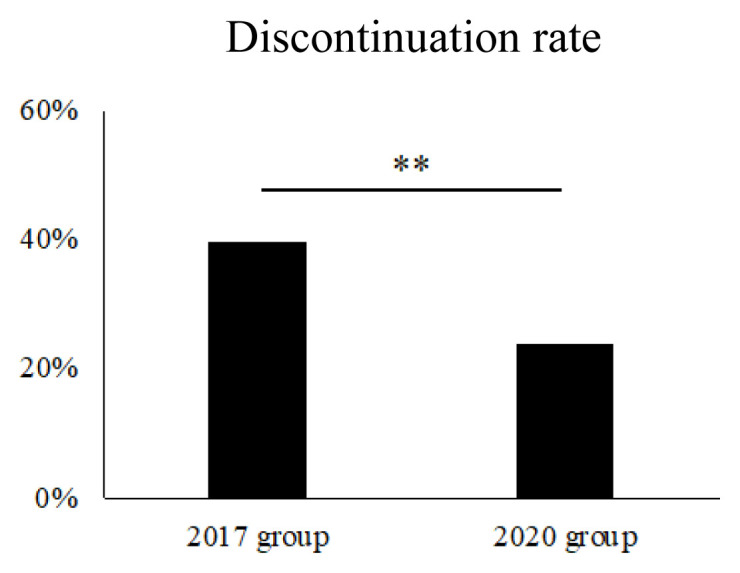
Rate of SGLT2 inhibitor discontinuation due to ADRs **p < 0.01.

Of the 323 patients, 99 patients discontinued SGLT2 inhibitors due to ADRs across the entire period. We compared the patients who were discontinued SGLT2 inhibitors between the 2017 group and the 2020 group (Figure 2; Table III). Notably, it revealed that discontinuations due to ADRs were significantly lower in the 2020 group (55 patients, 39.6% vs. 44 patients, 23.9%; *p* = 0.00335). Moreover, the 2020 group had higher median age, longer disease duration, lower body mass index and lower fasting C-peptide than the 2017 group.

To rule out the possibility that differences in treatment duration affected the results, we compared the treatment duration. The treatment duration was identifiable in 35 out of 99 patients (35.4%) in the entire period, 23 out of 55 patients (41.8%) in the 2017 group, and 12 out of 44 patients (27.3%) in the 2020 group, respectively. The median treatment duration was 9 months (1–22 months) in the 2017 group and 4 months (2–14.8 months) in the 2020 group (*p* = 0.601). Despite the limitation that treatment duration could be identified for less than half of the patients, there was no significant difference in treatment duration until discontinuation between the two groups.

Examining the cause of discontinuation, the most common was volume depletion in 22 patients (6.81%), followed by ketosis in 19 patients (5.88%), and urinary tract/genital infections in 18 patients (5.57%). A Comparison of the cause of discontinuation between the 2017 group and the 2020 group revealed significant reductions in urinary tract/genital infections (12 patients, 8.63% vs. 6 patients, 3.26%), ketosis (12 patients, 9.35% vs. 6 patients, 3.26%), and cerebral infarction (7 patients, 5.04% vs. 2 patients, 1.09%). Conversely, there was a significant increase in discontinuation due to frailty (0 patient, 0.00% vs. 7 patients, 3.80%) (Table IV).

**Figure 3:**
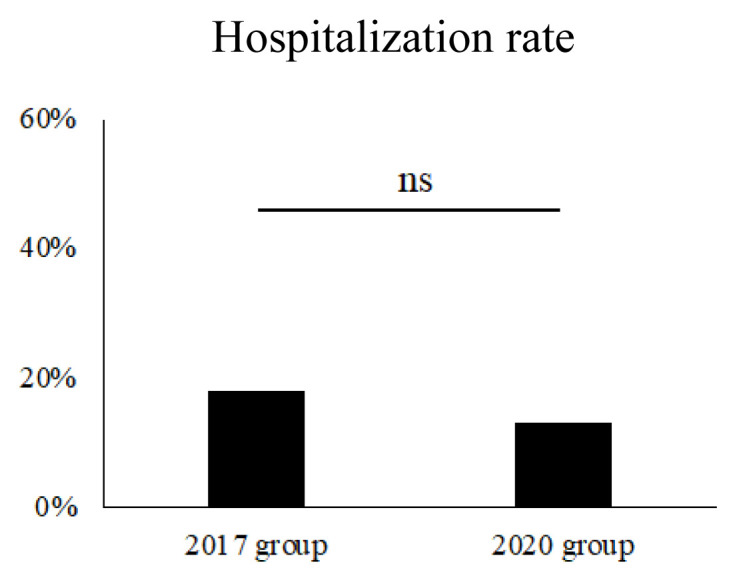
Rate of hospitalization due to ADRs ns, not significant.

Finally, we focused on severe ADRs and examined their changes over time (Table V; Figure 3). Severe ADRs were defined as hospitalizations potentially related to SGLT2 inhibitor ADRs, specifically those due to urinary tract/genital infections, ketosis including diabetic ketoacidosis (DKA), volume depletion including hyperosmolar hyperglycemic state (HHS), hypoglycemia, cerebral infarction, and gangrene with leg amputation. The highest incidence rates were 3.41% (11 patients) for volume depletion, 3.10% (10 patients) for urinary tract/genital infections, and 2.79% (9 patients) for ketosis. Comparing the 2017 group to the 2020 group, there was a nonsignificant but decreasing trend in the overall incidence of severe ADRs in the 2020 group (25 patients, 18.0% vs. 24 patients, 13.0%; *p* = 0.273). When examining the incidence of each severe ADR, only hospitalizations due to cerebral infarction showed a significant reduction. There were no significant changes in hospitalization due to urinary tract/genital infections, DKA, or volume depletion. HHS was only 2 patients in the 2017 group and 1 patient in the 2020 group. There were no deaths throughout the entire period.

## DISCUSSION

In this retrospective observational study, we evaluated changes over time in severe ADRs requiring discontinuation or hospitalization during SGLT2 inhibitor therapy. Patients with a history of SGLT2 inhibitor prescriptions were older and had lower HbA1c in the 2020 group (April 2020–March 2023) than in the 2017 group (April 2017–March 2020). This is suggested to be due to the increased prescribing of SGLT2 inhibitors to elderly patients as their safety was recognized and their efficacy in chronic heart failure and chronic kidney disease was reported, and the administration of these drugs increased not only for the purpose of improving HbA1c but also for the purpose of providing adjunctive benefits.

This study showed that discontinuations due to ADRs decreased in the 2020 group compared to the 2017 group, despite the increased age of patients receiving the drug. Specifically, discontinuations due to cerebral infarction, ketosis, and urinary tract/genital infections decreased, suggesting that proper use of the drug has spread due to alerts from academic societies and other sources. However, a comparison of the clinical backgrounds of patients who discontinued due to ADRs over time showed that they were older, had longer disease duration, and lower body mass index in the 2020 group than those in the 2017 group, with more discontinuations due to frailty. Continued caution is required when prescribing SGLT2 inhibitors to elderly patients at high risk of frailty. Furthermore, there was no significant decrease in ADRs-related hospitalization, including urinary tract infections, diabetic ketoacidosis, or volume depletion. This highlights the importance of continuing enlightenment activities regarding the ADRs of SGLT2 inhibitors.

Several recent meta-analyses have reported the safety of SGLT2 inhibitors compared with placebo (10, 11, 16). However, few studies have reported a direct comparison of ADRs associated with SGLT2 inhibitors across different time periods.

This study did have four limitations. The first is that the study included patients who were hospitalized for any reason and did not include outpatients, resulting in a biased population. In the DAHLIA, SAPPHIRE, and STELLA-LONG TERM studies of Japanese patients with type 2 diabetes, the mean age was 56.9–58.4 years, which is different from the population in this study, and the ADRs that resulted in discontinuation were 5.1% to 7.2%, clearly lower than those in the present study (17–19). Furthermore, even in the EMPA-ELDERLY study of patients 65 years of age and older, the incidence of ADRs was 12.9% and the number of cases of discontinuation due to ADRs was 4.6% (20), which were lower than those in the present study. These differences suggest that the population in this study may be at higher risk for ADRs. On the other hand, our hospital is one of the leading acute care hospitals in Osaka in terms of the number of emergency admissions, and it is likely that we capture a high rate of emergency admissions due to severe ADRs. The KAMOGAWA-A Study reported that the most common cause of discontinuation was polyuria/frequent urination, which would be expected to account for a high proportion of mild ADRs (21). In the present study, the most common cause of discontinuation was ketosis (19 patients, 5.88%), followed by urinary tract/genital infections (18 patients, 5.57%). Of these cases, 47.4% (9 patients) required hospitalization due to ketosis and 55.6% (10 patients) required hospitalization due to urinary tract/genital infections, indicating a high proportion of severe cases. Second, sample size was small because the study was conducted at only one institution. It is possible that the study was not sufficiently thorough. Third, around 2020, the COVID-19 infection epidemic has led to a decrease in the number of diabetic patients visit and a decrease in the number of emergency patients, which should be carefully evaluated in a time series comparison study. Finally, it was limitation with data collection. Although there was no significant difference in the duration of administration between the groups, nearly half of the patients had an unknown duration of administration, which may result in insufficient analysis.

Although our study was conducted at only one institution, we were able to clarify the current status of SGLT2 inhibitor ADRs in diabetes patients over a long period of time at a community-based acute care hospital. We believe that our study accurately reflects the reality of SGLT2 inhibitor ADRs in real-world clinical settings.

## Figures and Tables

**Table I:** Clinical characteristics

	All (n = 323)	2017 group (n = 139)	2020 group (n = 184)	*P value*
**Clinical characteristics**				
Male, n [%]	222 [68.7]	93 [66.9]	129 [70.1]	0.547
Age (years)	64 (54–72.5)	62 (53–69.5)	65.5 (55–75)	0.005
Duration of diabetes (years), (n = 231)	8 (3–15)	7 (3–15)	10 (2–17.3)	0.393
Type 1/type 2 diabetes, n [%]	2/321 [0.6/99.4]	0/139 [0.0/100.0]	2/182 [1.1/98.9]	0.508
Body mass index (kg/m^2^)	27.5 ± 6.1	28.0 ± 6.9	27.1 ± 5.5	0.209
Systolic blood pressure (mmHg)	131.8 ± 18.3	131.6 ± 20.3	132.0 ± 16.9	0.863
Diastolic blood pressure (mmHg)	78.9 ± 13.7	79.5 ± 13.8	78.5 ± 13.6	0.488
Chronic heart failure, n [%]	40 [12.4]	19 [13.7]	21 [11.4]	0.61
Hypertension, n [%]	198 [61.3]	80 [57.6]	118 [64.1]	0.25
Drinking, n [%]	103 [31.9]	43 [30.9]	60 [32.6]	0.81
Diaper wearing, n [%]	10 [3.1]	3 [2.2]	7 [3.8]	0.524
Decline in ADL, n [%]	24 [7.4]	8 [5.8]	16 [8.7]	0.394
Dementia, n [%]	27 [8.4]	9 [6.5]	18 [9.8]	0.317
**Laboratory data**				
HbA1c (%)	8.8 (7.4–10.6)	9.7 (7.9–11.4)	8.2 (7.2–9.9)	<0.001
eGFR (mL/min/1.73m^2^)	72.7 (58.2–86.8)	71.2 (58.4–86.3)	73.6 (57.7–87.8)	0.515
UACR (mg/gCr), (n = 189)	27 (14.3–89.6)	25.9 (13.2–84.5)	27.2 (16.5–99.1)	0.573
Fasting C-peptide (ng/mL), (n = 208)	2.2 ± 1.4	2.1 ± 1.2	2.3 ± 1.6	0.294
Hematocrit (%)	43.0 ± 5.5	43.5 ± 4.8	42.7 ± 6.0	0.178
Uric acid (mg/dL), (n = 198)	5.5 (4.6–6.7)	5.3 (4.7–6.4)	5.8 (4.6–6.8)	0.143
**Complications**				
Retinopathy (NDR/SDR/PPDR/PDR), n [%], (n = 219)	150/41/19/9 [68.5/18.7/8.7/4.1]	72/26/10/4 [64.3/23.2/8.9/3.6]	78/15/9/5 [72.9/14.0/8.4/4.7]	0.363
Nephropathy (stage 1/2/3/4/5), n [%], (n = 197)	105/62/24/6/0 [53.3/31.5/12.2/3.0/0.0]	59/32/14/2/0 [55.1/29.9/13.1/1.9]	46/30/10/4/0 [51.1/33.3/11.1/4.4]	0.694
Cardiovascular disease, n [%]	43 [13.3]	17 [12.2]	26 [14.1]	0.741
Cerebral infarction, n [%]	21 [6.5]	8 [5.8]	13 [7.1]	0.82

ADL, activities of daily living; HbA1c, glycated hemoglobin; eGFR, estimated glomerular filtration rate; UACR, urinary albumin creatinine ratio; NDR, normal diabetic retinopathy; SDR, simple diabetic retinopathy; PPDR, pre-proliferative diabetic retinopathy; PDR, proliferative diabetic retinopathy.

**Table II:** Comparisons of medications

	All (n = 323)	2017 group (n = 139)	2020 group (n = 184)	*P value*
**Antidiabetic**				
Insulin, n [%]	71 [22.0]	31 [22.3]	40 [21.7]	1.000
Sulfonylurea, n [%]	68 [21.1]	26 [18.7]	42 [22.8]	0.410
Glinide, n [%]	46 [14.2]	17 [12.2]	29 [15.8]	0.423
Biguanide, n [%]	168 [52.0]	73 [52.5]	95 [51.6]	0.911
Thiazolidine, n [%]	46 [14.2]	21 [15.1]	25 [13.6]	0.749
α-Glucosidase inhibitor, n [%]	35 [10.8]	15 [10.8]	20 [10.9]	1.000
DPP-4 inhibitor, n [%]	214 [66.3]	89 [64.0]	125 [67.9]	0.478
GLP-1 receptor agonist, n [%]	38 [11.8]	14 [10.1]	24 [13.0]	0.508
**Antihypertensive**				
ACE inhibitor or ARB, n [%]	143 [44.3]	55 [39.6]	88 [47.8]	0.143
Calcium antagonist, n [%]	131 [40.6]	52 [37.4]	79 [42.9]	0.360
Beta-blocker, n [%]	48 [14.9]	18 [12.9]	30 [16.3]	0.433
Alpha-blocker, n [%]	8 [2.5]	4 [2.9]	4 [2.2]	0.729
Diuretic, n [%]	49 [15.2]	20 [14.4]	29 [15.8]	0.757
**Others**				
Steroid, n [%]	14 [4.3]	4 [2.9]	10 [5.4]	0.409
Immunosuppressant, n [%]	2 [0.6]	1 [0.7]	1 [0.5]	1.000

DPP-4, dipeptidy-l peptidase-4; GLP-1, Glucagon-Like Peptide-1; ACE, angiotensin-converting enzyme; ARB, angiotensin II receptor blocker.

**Table III:** Comparison of the discontinued patients between the 2017 group and the 2020 group

	2017 group (n = 55)	2020 group (n = 44)	*P value*
**Clinical characteristics**			
Male, n [%]	30 [54.5]	29 [65.9]	0.305
Age (years)	62 (54–72)	74 (62–78.3)	0.002
Duration of diabetes (years), (n = 65)	9.8 ± 8.1	18.7 ± 13.0	0.001
Type 1 diabetes, n [%]	0 [0.0]	1 [2.3]	0.444
Body mass index (kg/m^2^)	27.6 ± 7.7	24.1 ± 5.5	0.012
Systolic blood pressure (mmHg)	129.8 ± 23.2	132.2 ± 18.0	0.581
Diastolic blood pressure (mmHg)	76.1 ± 14.7	76.5 ± 11.7	0.885
Chronic heart failure, n [%]	8 [14.5]	5 [11.4]	0.768
Hypertension, n [%]	37 [67.3]	28 [63.6]	0.832
Drinking, n [%]	15 [27.3]	11 [25.0]	0.823
Diaper wearing, n [%]	3 [2.2]	7 [3.8]	0.524
Decline in ADL, n [%]	6 [10.9]	11 [25.0]	0.106
Dementia, n [%]	5 [9.1]	11 [25.0]	0.052
**Laboratory data**			
HbA1c (%)	9.0 (7.8–11.2)	8.4 (6.9–10.1)	0.168
eGFR (mL/min/1.73m^2^)	65.3 (48.8–83.6)	71.5 (41.9–91.5)	0.770
UACR (mg/gCr), (n = 55)	151.8 ± 355.4	338.1 ± 1177.3	0.391
Fasting C-peptide (ng/mL), (n = 68)	1.84 (1.22–2.45)	1.15 (0.82–2.23)	0.022
Hematocrit (%)	42.1 ± 4.9	39.7 ± 7.4	0.056
Uric acid (mg/dL), (n = 59)	5.7 ± 1.2	6.0 ± 2.0	0.464
**Complications**			
Retinopathy (NDR/SDR/PPDR/PDR), n [%], (n = 63)	22/10/3/2 [59.5/27.0/8.1/5.4]	15/5/3/3 [57.7/19.2/11.5/11.5]	0.703
Nephropathy (stage 1/2/3/4/5), n [%], (n = 60)	20/9/5/2/0 [55.6/15.0/13.9/5.6/0.0]	10/9/2/3/0 [41.7/37.5/8.3/12.5/0.0]	0.496
Cardiovascular disease, n [%]	7 [12.7]	6 [13.6]	0.762
Cerebral infarction, n [%]	6 [10.9]	6 [13.6]	1.000

**Table IV:** Comparison of the cause of discontinuation between the 2017 group and the 2020 group

The cause of discontinuation	2017 group (n = 139)	2020 group (n = 184)	*P value*
Urinary tract/genital infections, n [%]	12 [8.63]	6 [3.26]	0.049
Polyuria/pollakiuria, n [%]	2 [1.44]	0 [0.00]	0.184
Ketosis, n [%]	13 [9.35]	6 [3.26]	0.030
Diabetic ketoacidosis, n [%]	3 [2.16]	3 [1.63]	1.000
Volume depletion, n [%]	10 [7.19]	12 [6.52]	0.827
Hypoglycemia, n [%]	0 [0.00]	2 [1.09]	0.508
Cerebral infarction, n [%]	7 [5.04]	2 [1.09]	0.042
Lower limb gangrene, n [%]	2 [1.44]	1 [0.54]	0.579
Frailty, n [%]	0 [0.00]	7 [3.80]	0.021
Skin complications, n [%]	0 [0.00]	1 [0.54]	1.000
Fracture, n [%]	0 [0.00]	1 [0.54]	1.000
Renal disorder, n [%]	2 [1.44]	2 [1.09]	1.000
Hepatic disorder, n [%]	0 [0.00]	1 [0.54]	1.000
Cardiovascular disease, n [%]	1 [0.72]	0 [0.00]	0.430
Pancreatitis, n [%]	1 [0.72]	0 [0.00]	0.430
Others, n [%]	2 [1.44]	0 [0.00]	0.184

**Table V:** Comparison of the severe ADRs between the 2017 group and the 2020 group

The cause of hospitalization	2017 group (n = 139)	2020 group (n = 184)	*P value*
Urinary tract/genital infections, n [%]	6 [4.32]	4 [2.17]	0.337
Ketosis, n [%]	3 [2.16]	6 [3.26]	0.737
Diabetic ketoacidosis, n [%]	3 [2.16]	3 [1.63]	1.000
Volume depletion, n [%]	4 [2.88]	7 [3.80]	0.763
Hypoglycemia, n [%]	0 [0.00]	1 [0.54]	1.000
Cerebral infarction, n [%]	7 [5.04]	2 [1.09]	0.042
Lower limb gangrene, n [%]	2 [1.44]	1 [0.54]	0.579
